# Draft genome sequence of *Rhodococcus rhodochrous* strain ATCC 17895

**DOI:** 10.4056/sigs.4418165

**Published:** 2013-10-05

**Authors:** Bi-Shuang Chen, Linda G. Otten, Verena Resch, Gerard Muyzer, Ulf Hanefeld

**Affiliations:** 1 Delft University of Technology, Department of Biotechnology, Biocatalysis group, Gebouw voor Scheikunde, the Netherlands; 2 University of Amsterdam, Department of Aquatic Microbiology, Institute for Biodiversity and Ecosystem Dynamics, the Netherlands

**Keywords:** *Rhodococcus rhodochrous*, *Rhodococcus erythropolis*, biocatalysis, genome

## Abstract

*Rhodococcus rhodochrous* ATCC 17895 possesses an array of mono- and dioxygenases, as well as hydratases, which makes it an interesting organism for biocatalysis. *R. rhodochrous* is a Gram-positive aerobic bacterium with a rod-like morphology. Here we describe the features of this organism, together with the complete genome sequence and annotation. The 6,869,887 bp long genome contains 6,609 protein-coding genes and 53 RNA genes. Based on small subunit rRNA analysis, the strain is more likely to be a strain of *Rhodococcus erythropolis* rather than *Rhodococcus rhodochrous*.

## Introduction

The genus *Rhodococcus* comprises genetically and physiologically diverse bacteria, known to have a broad metabolic versatility, which is represented in its clinical, industrial and environmental significance. Their large number of enzymatic activities, unique cell wall structure and suitable biotechnological properties make *Rhodococcus* strains well-equipped for industrial uses, such as biotransformation and the biodegradation of many organic compounds. In the environmental field, the ability of *Rhodococcus* to degrade trichloroethene [1], haloalkanes [2-4], and dibenzothiophene (DBT) [5] is reported. Furthermore, its potential for petroleum desulfurization is known [5].

*Rhodococcus rhodochrous* strains are ubiquitous in nature. They possess an array of mono- and dioxygenases, as well as hydratases, which make them an interesting organism for biocatalysis [6]. One example would be the recently reported regio-, diastereo- and enantioselective hydroxylation of unactivated C-H bonds [7] which remains a challenge for synthetic chemists, who often rely on differences in the steric and electronic properties of bonds to achieve regioselectivity [8]. Furthermore, most *Rhodococcus* strains harbor nitrile hydratases [9-11], a class of enzymes used in the industrial production of acrylamide and nicotinamide [12] while other strains are capable of transforming indene to 1,2-indandiol, a key precursor of the AIDS drug Crixivan [13]. In another recent example, *R. rhodochrous* ATCC BAA-870 was used for the biocatalytic hydrolysis of β-aminonitriles to β-amino-amides [14]. One example for a rather rarely investigated reaction would be the biocatalytic hydration of 3-methyl- or 3-ethyl-2-butenolide from the corresponding (*R*)-3-hydroxy-3-alkylbutanolide, a phenomenon observed in resting cells of *Rhodococcus rhodochrous* strain ATCC 17895 [15].

In order to obtain a comprehensive understanding of its high ability for biodegradation and biotransformation [16], the genome of *R. rhodochrous* strain ATCC 17895 was sequenced. To the best of our knowledge, no complete genome sequence of this organism can be found in the literature. Here we present a summary, classification and a set of features for *R. rhodochrous* strain ATCC 17895 together with the description of the genomic sequencing and annotation.

## Classification and features

Bacteria from the *Rhodochrous* group are taxonomically related to the genera *Nocardia* and *Mycobacterium*. In 1977 Goodfellow and Alderson proposed the genus *Rhodococcus* to be assigned to this group [17]. This assignment is due to the overlapping characteristics with *Nocardia* and *Mycobacterium* that were studied in morphological, biochemical, genetic, and immunological studies [18]. *R. rhodochrous* strain ATCC 17895 was previously deposited as *Nocardia erythropolis* [19] and *Rhodococcus erythropolis* [17].

When incubated with fresh nutrient medium, *R. rhodochrous* grows as rod-shaped cells [20]. Furthermore cells are described to be Gram-positive actinomycetes with a pleomorphic behavior often forming a primary mycelium that soon fragments into irregular elements [21,22]. It is known to be a facultative aerobe, non-motile and may be partially acid-fast. Production of endospores or conidia has not been reported, but for some strains a few feeble aerial hyphae are observed [23,24]. The optimal growth temperature reported is 26 ^o^C on standard culture media. After initially growing sparsely, *R. rhodochrous* strain ATCC 17895 forms organized lumps on the agar surface, leading to the growth of dry opaque, pale orange, concentrically ringed colonies (Figure 1A and 1B). Usually growth is observed within 3 to 4 days.

**Figure 1A:**
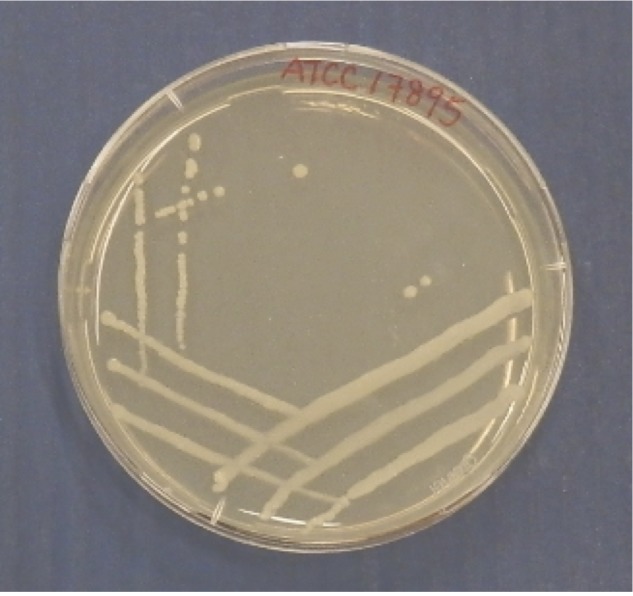
Characteristic of strain ATCC 17895 on nutrient agar plate after 72 h

**Figure 1B:**
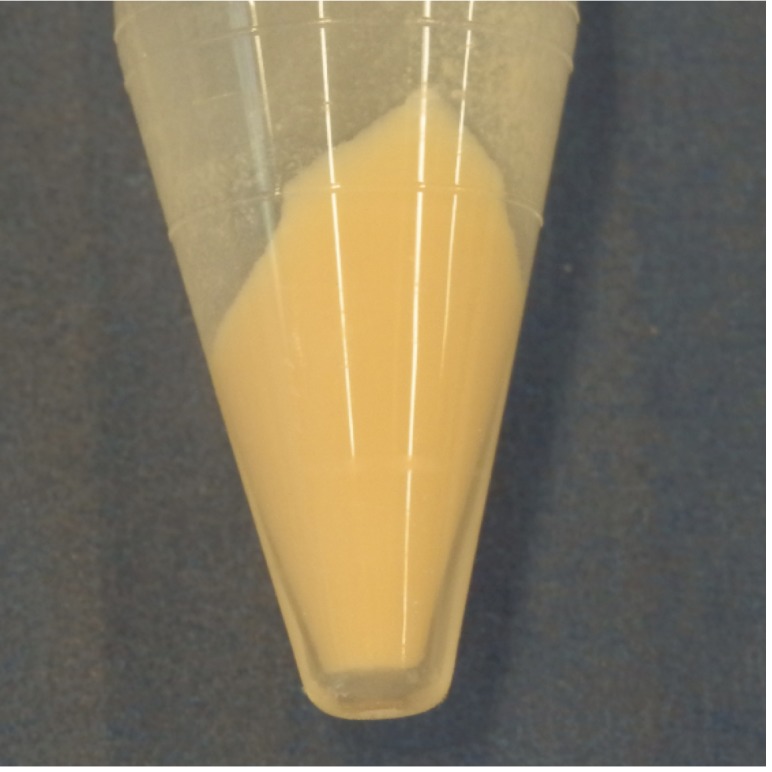
Harvested pale orange cells incubated with fresh nutrient medium after 72 h.

**Figure 2:**
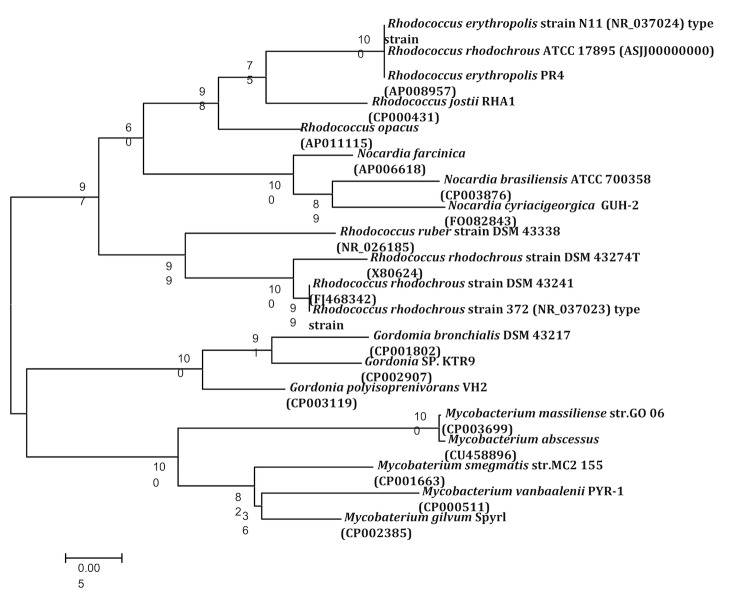
Phylogenetic tree based on the 16S rRNA sequence highlighting the phylogenetic position of *Rhodococcus rhodochrous* strain ATCC 17895 relative to other type strains within the genus *Rhodococcus*. Genbank accession numbers are indicated in parentheses. Sequences were aligned using CLUSTALW, and phylogenetic inferences were obtained using the neighbor-joining method within the MEGA v5 software [38]. Numbers at the nodes are percentages of bootstrap values obtained by repeating the analysis 1,000 times to generate a majority consensus tree. The scale bar indicates 0.005 nucleotide change per nucleotide position.

*R. rhodochrous* strains are known to produce acid from glycerol, sorbitol, sucrose and trehalose, but not from adonitol, arabinose, cellobiose, galactose, glycogen, melezitose, rhamnose or xylose. The cell wall peptidoglycan incorporates meso-diaminopimelic acid, arabinose and galactose (wall type IV) [25]. The bacterium is urease and phosphatase positive. The important characteristics of the strain based on literature descriptions are summarized in Table 1. On the basis of 16S rRNA gene sequencing the strain belongs to the genus *Rhodococcus* within class *Actinobacteria*, *Rhodococcus erythropolis* PR4 and *Rhodococcus erythropolis* strain N11 are its closest phylogenetic neighbors (Figure 2).

**Table 1:** Classification and general features of *Rhodococcus rhodochrous* ATCC 17895 according to the MIGS recommendations [26]

**MIGS ID**	**Property**	**Term**	**Evidence code**
		Domain *Bacteria*	TAS [27]
		Phylum *Actinobacteria*	TAS [28]
		Class *Actinobacteria*	TAS [29]
		Subclass *Actinobacteridae*	TAS [29,30]
		Order *Actinomycetales*	TAS [29-32]
		Suborder *Corynebacterineae*	TAS [29,30]
		Family *Nocardiaceae*	TAS [29,30,32,33]
		Genus *Rhodococcus*	TAS [32,34]
		Species *Rhododoccus rhodochrous*	TAS [32,35,36]
		Strain ATCC17895	
	Gram stain	Positive	TAS [17]
	Cell shape	Rod-shaped	TAS [20]
	Motility	Non-motile	TAS [17]
	Sporulation	Non-sporulating	TAS [17]
	Temperature range	Mesophile	TAS [17]
	Optimum temperature	26 ^o^C	TAS [19]
MIGS-6.3	Salinity	Not reported	NAS
MIGS-22	Oxygen requirement	Aerobe	TAS [17]
	Carbon source	fructose, glucose, mannose, sucrose	TAS [17]
	Energy source	butyrate, fumarate, propionate	TAS [17]
MIGS-6	Habitat	Marine, Aquatic	TAS [17]
MIGS-15	Biotic relationship	Free-living	TAS [37]
MIGS-14	Pathogenicity	Not reported	NAS
	Biosafety level	1	TAS [19]
	Isolation	Pacific Ocean seawater	TAS [37]
MIGS-4	Geographic location	Canada	TAS [37]
MIGS-5	Sample collection time	Not reported	NAS
MIGS-4.1	Latitude	Not reported	NAS
MIGS-4.2	Longitude	Not reported	NAS
MIGS-4.3	Depth	Not reported	NAS
MIGS-4.4	Altitude	Not reported	NAS

Evidence codes – IDA: Inferred from Direct Assay (first time in publication); TAS: Traceable Author Statement (i.e., a direct report exists in the literature); NAS: Non-traceable Author Statement (i.e., not directly observed for the living, isolated sample, but based on a generally accepted property for the species, or anecdotal evidence). These evidence codes are from the Gene Ontology project. If the evidence code is IDA, then the property was directly observed by one of the authors or an expert mentioned in the acknowledgments.

## Genome sequencing information

### Genome project history

This organism was selected for sequencing on the basis of its common use for a wide range of biotransformation, such as steroid modification, enantioselective synthesis, the production of amides from nitriles [6,39,40], and its interesting hydration capabilities [15]. The complete genome obtained in this study was sequenced in October 2012 and has been deposited at GenBank under accession number ASJJ00000000 consisting of 423 contigs (≥300 bp) and 376 scaffold (≥300 bp). The version described in this paper is version ASJJ01000000. Sequencing was performed by BaseClear BV (Leiden, the Netherlands) and initial automatic annotation by Institute for Biodiversity and Ecosystem Dynamics (Amsterdam). A summary of the project information is shown in Table 2.

**Table 2:** Genome sequencing project information

**MIGS ID**	**Characteristic**	**Details**
MIGS-28	Libraries used	One Illumina paired-end library, 50 cycles
MIGS-29	Sequencing platform	Illumina HiSeq 2000
MIGS-31.2	Sequencing coverage	50 ×
MIGS-31	Finishing quality	Permanent draft
MIGS-30	Assemblers	CLCbio Genomics Workbench version 5.5.1
MIGS-32	Gene calling method	RAST
	BioProject	PRJNA201088
	GenBank ID	ASJJ00000000
	GenBank date of release	September 23, 2013
MIGS-13	Source material identifier	ATCC 17895
	Project relevance	Biotechnology

### Growth conditions and DNA isolation

*Rhodococcus rhodochrous* ATCC 17895 was grown on nutrient medium [8.0 g nutrient broth (BD cat. 234000) in 1000 mL demi water] at pH 6.8 and 26 ^o^C with orbital shaking at 180 rpm as recommended by ATCC. Extraction of chromosomal DNA was performed by using 50 mL of overnight culture, centrifuged at 4 ^o^C and 4,000 rpm for 20 min and purified using the following method [41]. Then, 100 mg wet cells were transferred to a microcentrifuge tube and washed three times with 0.5 mL potassium phosphate buffer (0.1 M, pH 6.2). The resulting cell pellet was resuspended in 564 µL Tris-HCl buffer (10 mM) containing 1 mM EDTA (pH 8.0) and 10 µg lysozyme and incubated at 37 ^o^C for 2 h. Next, Proteinase K (3 µL of 20 mg/mL stock), DNase-free RNase (2 µL of 10 mg/mL stock), SDS (50 µL of 20% w/v stock) were added and the cell suspension was incubated at 50 ^o^C for 3 h followed by the addition of 5 M NaCl (100 µL) and incubation at 65 ^o^C for 2 min. After addition of 80 µL of CTAB/NaCl solution (10% w/v hexadecyl trimethyl ammonium bromide in 0.7 M NaCl) incubation at 65 ^o^C for 10 min was performed. The cell lysate was twice extracted with phenol/chloroform/isoamyl alcohol (25:24:1) and the aqueous layer was separated after centrifugation at 14,000 rpm for 15 min. The DNA was precipitated with 0.7 volumes isopropanol and dissolved in sterile water for genome sequencing. The quality and quantity of the extracted DNA was evaluated by 0.8% (w/v) agarose gel electrophoresis to obtain good quality DNA, with an OD260:280 ratio of 1.8-2, and as intact as possible.

### Genome sequencing and assembly

Genomic DNA libraries for the Illumina platform were generated and sequenced at BaseClear BV (Leiden, The Netherlands). High-molecular weight genomic DNA was used as input for library preparation using the Illumina TruSeq DNA library preparation kit (Illumina). Briefly, the gDNA was fragmented and subjected to end-repair, A-tailing, ligation of adaptors including sample-specific barcodes and size-selection to obtain a library with median insert-size around 300 bp. After PCR enrichment, the resultant library was checked on a Bioanalyzer (Agilent) and quantified. The libraries were multiplexed, clustered, and sequenced on an Illumina HiSeq 2000 with paired-end 50 cycles protocol. The sequencing run was analyzed with the Illumina CASAVA pipeline (v1.8.2). The raw sequencing data produced was processed removing the sequence reads which were of too low quality (only "passing filter" reads were selected) and discarding reads containing adaptor sequences or PhiX control with an in-house filtering protocol. The quality of the FASTQ sequences was enhanced by trimming off low-quality bases using the “Trim sequences” option of the CLC Genomics Workbench version 5.5.1. The quality filtered sequence reads were puzzled into a number of contig sequences using the “De novo assembly” option of the CLC Genomics Workbench version 5.5.1. Subsequently the contigs were linked and placed into scaffolds or supercontigs with SSPACE premium software v2.3 [42]. The orientation, order and distance between the contigs were estimated using the insert size between the paired-end reads. Finally, the gapped regions within the scaffolds were (partially) closed in an automated manner using GapFiller v 1.10 [43].

### Genome annotation

Genes were identified and annotated using RAST (Rapid Annotations based on Subsystem Technology) [44]. The translated CDSs were used to search the National Center for Biotechnology Information (NCBI) nonredundant (nr) database, Pfam, KEGG, and COG databases. Additional gene prediction analysis and functional annotation were performed within the Integrated Microbial Genomes Expert Review (IMG-ER) platform [45].

## Genome properties

The genome size is around 6,869,887 bp. The G+C percentage determined from the genome sequence is 62.29%, which is similar to the value of its closest sequenced neighbor *R. erythropolis* PR4, determined by Sekine M [46]. The genomic information of strain PR4 was deposited to GenBank, but was not publicly available until very recent. From the genome sequence of strain ATCC 17895, there are 6,662 predicted genes, of which 6,609 are protein-coding genes, and 53 are RNA genes. A total of 5,186 genes (77.8%) are assigned a putative function. The remaining genes are annotated as either hypothetical proteins or proteins of unknown functions. The properties and statistics of the genome are summarized in Table 3 and the distribution of genes into COGs functional categories is presented in Table 4. The number and percentage of genes in different COG categories is equivalent to the closely related *R. erythropolis* PR4 and *R. jostii* RHA1, showing that most genes have been annotated, even though the genome was not fully closed.

**Table 3:** Genome statistics

**Attribute**	**Value**	**% of Total**
Genome size (bp)	6,869,887	100.00
DNA coding region (bp)	6,017,668	87.63
DNA G + C content (bp)	4,279,255	62.29
Number of replicons	1	
Extrachromosomal elements (plasmid)	0	
Total genes	6,662	100.00
RNA genes	53	0.80
rRNA operons	3	0.05
Protein-coding genes	6,609	99.20
Pseudogenes	0	
Genes in paralog clusters	5,469	82.09
Genes assigned to COGs	4,751	71.31
Genes assigned Pfam domains	5,132	77.03
Genes with signal peptides	305	4.58
CRISPR repeats	0	

**Table 4:** Number of genes associated with the general COG functional categories.

**Code**	**Value**	**% age**	**Description**
J	194	3.63	Translation, ribosomal structure and biogenesis
A	5	0.09	RNA processing and modification
K	597	11.16	Transcription
L	155	2.90	Replication, recombination and repair
B	1	0.02	Chromatin structure and dynamics
D	42	0.79	Cell cycle control, mitosis and meiosis
V	88	1.64	Defense mechanisms
T	241	4.50	Signal transduction mechanisms
M	198	3.70	Cell wall/membrane biogenesis
N	4	0.07	Cell motility
U	37	0.69	Intracellular trafficking and secretion
O	143	2.67	Posttranslational modification, protein turnover, chaperones
C	364	6.80	Energy production and conversion
G	339	6.34	Carbohydrate transport and metabolism
E	460	8.60	Amino acid transport and metabolism
F	103	1.93	Nucleotide transport and metabolism
H	187	3.5	Coenzyme transport and metabolism
I	427	7.98	Lipid transport and metabolism
P	323	6.04	Inorganic ion transport and metabolism
Q	327	6.11	Secondary metabolites biosynthesis, transport and catabolism
R	711	13.29	General function prediction only
S	404	7.55	Function unknown
-	1911	28.69	Not in COGs

As is obvious from Figure 2, the 16S rRNA of this *R. rhodochrous* strain is much closer to *R. erythropolis* than to *R. rhodochrous*. Also *R. erythropolis* PR4 is the closest neighbor of the currently sequenced organism. Furthermore, certain genes mentioned by Gürtler et al. to be part of *R. erythropolis* strains, but not to be present in *R. rhodochrous* [47], are all present in the genome. Therefore, as recommended by Gürtler *et al*., we propose that this organism should be reclassified as a strain of *Rhodococcus erythroplis* (*Rhodococcus erythroplis* ATCC 17895).

### Biocatalytic properties

Since we are interested in the biocatalytic properties of this organism, we looked at enzymes known to be abundant in *Rhodococcus* strains. There are 27 different mono- and dioxygenases annotated in the genome, which is similar to the number in the closely related *R. erythropolis* PR4. And, as expected, there are 2 ureases and more than 10 phosphatases in the genome. Furthermore, there is a full nitrile metabolizing operon present, comprising nitrile hydratase, regulators, amidase and aldoxime dehydratase. Although this organism is not a catabolic powerhouse like *Rhodococcus sp.* RHA1 [48], which was isolated from a polluted soil, there are numerous genes coding for proteins involved in producing amino acids, cofactors and lipids. For many of these proteins there are several copies of genes with similar function. This shows the versatility of this organism, like most members of its species. The various enzymes found by this genomic annotation can be used as a starting point to exploit this organism for biocatalytic operation, for instance, the rarely investigated biocatalytic hydration [15,49], and the hydroxylation of unactivated C-H bonds [7], which remains a major challenge for synthetic chemists.
